# Construction of a TTT-η Diagram of High-Refractive Polyurethane Based on Curing Kinetics

**DOI:** 10.3390/polym13203474

**Published:** 2021-10-10

**Authors:** Shidi Huang, Guiming Zhang, Weiping Du, Huifang Chen

**Affiliations:** 1 School of Ecological Technology and Engineering, Shanghai Institute of Technology, Shanghai 201418, China; sdhuang@sit.edu.cn; 2 College of Materials Science and Engineering, Donghua University, Shanghai 201620, China; gmzhang1688@163.com (G.Z.); duweiping@dhu.edu.cn (W.D.); 3 State Key Laboratory for Modification of Chemical Fibers and Polymer Materials, Donghua University, Shanghai 201620, China

**Keywords:** polyurethane, curing kinetics, TTT diagram, TTT-η diagram

## Abstract

A time–temperature–transformation–viscosity (TTT-η) diagram can reflect changes in the physical states of a resin, which take on significance for the study of the curing process of polyurethane resin lenses. Coupling the differential scanning calorimetry (DSC) test, the curing kinetic parameters of 1,4-bis(isocyanatomethyl)cyclohexane (H_6_XDI)/2,3-bis((2-mercaptoethyl)thio)-1-propanethiol (BES) polyurethane system were obtained. By phenomenological modeling, the relationships between degree, temperature, and time were obtained. An isothermal DSC test was carried out at 423 K. Based on the DiBenedetto equation, the relationships between glass transition temperature, degree of cure, and time were obtained, and the glass transition temperature was thus correlated with temperature and time. The gelation time at different temperatures was measured by rotary rheometry, and the relationship between gelation time and gelation temperature was established. The time–temperature–transformation (TTT) diagram of H_6_XDI/BES system was constructed accordingly. Subsequently, a six-parameter double Arrhenius equation was used as the basis for the rheological study. The viscosity was examined during the curing process. The TTT-η diagram was obtained, which laid the theoretical foundation for the optimization and setting of processing parameters.

## 1. Introduction

With the rapid development in science and technology, the number of people with myopia is increasing significantly, which promotes the demand for resin lenses. Polycarbonate (PC), polymethyl methacrylate (PMMA), episulfide resin, etc., are popular materials for lenses. Polyurethane resin has excellent optical performance and has been used for producing high-end resin lenses. Compared with traditional lenses, polyurethane lenses are called high-refractive lenses because their refractive index is commonly greater than 1.60. Domestic optical polyurethane producers generally lack core technology and theoretical guidance. Therefore, the curing mechanism of the H_6_XDI/BES system and the time–temperature–transformation–viscosity (TTT-η) diagram play a pivotal role in studying the curing of polyurethane resin lenses.

A TTT-η diagram can reflect changes in the physical states of a resin during the curing process. Phenomenological modeling and mechanism methods are the main ways to study the curing kinetics of a resin. Phenomenological modeling is more frequently used than the mechanism method [1]. The relationships between temperature, time, glass transition temperature, viscosity, etc., can be indicated by the TTT-η diagram. Zhang et al. [2] investigated the biomass epoxy resin system with abietic acid anhydride as the curing agent. Isothermal and heterotherm differential scanning calorimetry (DSC) were adopted to study the curing kinetics of the system. The relevant parameters were calculated based on phenomenological modeling, and the gelation time of the system was measured by rheometry. Then, a time–temperature–transformation (TTT) diagram was drawn, which provided instructions for the production technique. Zhang et al. [3] investigated the TTT diagram of BA9913 resin. The curing parameters were obtained by DSC in combination with phenomenological modeling. The gelation time of the resin system was determined by measuring the storage and loss moduli, and the production technique of the T300/BA9913 system was optimized according to the TTT diagram. Teil et al. [4] obtained the TTT diagram of an anhydride system, evaluated the gelation point, and obtained the gelation curve of the system by the rheological method. The curing kinetic parameters were obtained based on DSC, and new kinetic equations were constructed by optimizing the kinetic equations based on the Sesta–Berggren relation. Restrepo-Zapata et al. [5] obtained the TTT diagram of an aliphatic epoxy system using autocatalysis modeling. According to the DSC data, the relevant parameters of the system were obtained based on the Kissinger equation. Besides, the DiBenedetto equation was used to determine the relation between glass transition temperature and degree of cure, and the TTT-η diagram was constructed on the basis of the TTT diagram.

The H_6_XDI/BES system is a relatively new system in the field of high-refraction lenses in worldwide lens manufacture. Little research has been conducted on the lens production process of this system. The TTT-η diagram could be considered as a useful tool to guide and instruct the practical curing process. In this study, phenomenological modeling was chosen to investigate the curing kinetics of the H_6_XDI/BES resin system. In combination with the viscosity changes in the system during the curing process, the TTT-η diagram was constructed.

## 2. Materials and Methods

### 2.1. Chemicals

1,4-bis(isocyanatomethyl)cyclohexane (H_6_XDI, 98%) was purchased from Wanhua Chemical Group Co., Ltd. (Shandong, China) 2,3-Bis((2-mercaptoethyl)thio)-1-propanethiol (BES, 99%) was provided by Mitsui Chemicals, Inc. (Tokyo, Japan) Phosphate mold-release agent was provided by Shanghai Longxu Chemical Co., Ltd. (Shanghai, China). Dibutyltin dichloride (99%) was purchased from Macklin Inc. (Rochelle, IL, USA). All the chemicals were used without further purification.

### 2.2. Sample Preparation

The polythiourethane thermoset system was prepared by reacting BES with H_6_XDI at the ratio of functional groups of 1:1, using dibutyltin dichloride as the catalyst with a content of 150 mg L^−1^. After adding 0.1 mL of phosphate mold-release agent, the bottle was cooled to 0 °C, and the sample was stirred in a nitrogen atmosphere. Then, the sample was degassed under vacuum for 30 min and filtered by a 1 μm filter.

### 2.3. Characterization

#### 2.3.1. Differential Scanning Calorimetry (DSC)

A differential scanning calorimeter (TA, US) was used to perform non-isothermal and isothermal measurements for the BES/H_6_XDI sample under a nitrogen atmosphere at a flow rate of 50 mL/min. The H_6_XDI/BES system was incubated at 423 K for 25 min, 30 min, or 35 min, and then cooled to 298 K, followed by heating to 523 K at a rate of 10 K/min in order to obtain the residual enthalpy of the resin system.

For the non-isothermal test, the sample was heated from 273 K to 523 K at a heating rate of 3 K/min, 6 K/min, 9 K/min, and 12 K/min, respectively. To obtain the Tg_0_ and Tg_∞_ of the system, the sample was first heated from 273 K to 523 K at a heating rate of 5 K/min, and then cooled to 303 K naturally, followed by a second heating to 523 K at the same heating rate.

#### 2.3.2. Rheological Measurements

Rheological measurements were conducted using a HAAKE MARS 60 rheometer (Thermo Fisher Scientific Inc., Germany) equipped with parallel plates (35 mm in diameter). The distance between the plates was 1 mm. The temperature was set at 423 K, 433 K, and 443 K, respectively. The frequency was fixed at 1 Hz, and the shear stress was 30 Pa. Gelation time for isothermal curing was determined as the point where storage (G’) and loss modulus (G”) intersected.

## 3. Results and Discussion

### 3.1. Construction of TTT Diagram

#### 3.1.1. Determination of Tg_0_ and Tg_∞_

**Figure 1:**
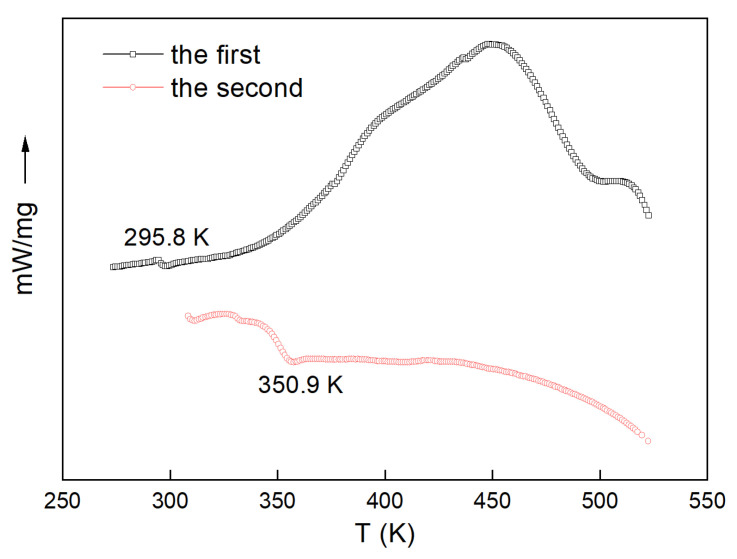
The first and second DSC scanning curves for the H_6_XDI/BES system.

DSC scanning was performed twice for the H_6_XDI/BES system, the results of which are shown in Figure 1. In the first DSC scanning, a baseline shift occurred at the point of 295.8 K, which was regarded as Tg_0_. The second DSC scanning was performed after the system was subjected to the temperature changes during the first scanning. The resultant curve indicates that the system was cured completely. The point where baseline shift occurred was at 350.9 K, regarded as Tg_∞_.

#### 3.1.2. Determination of Iso–Curing Lines

Two hypotheses were made based on the resulting DSC data [6]: (1) The heat detected is exclusively from the H_6_XDI/BES system, and the integral of exothermic peaks in the DSC curves positively correlates with the total heat released from the H_6_XDI/BES system; (2) The heat flow rate of the system correlates positively with the curing rate, and there is an equation as follows:

(1) $$\frac{d\alpha }{dt}=\frac{dH}{dt}*\frac{1}{\Delta H}$$

where ΔH is the total heat released from the H_6_XDI/BES system; $\frac{d\alpha }{dt}$ is the curing rate; and $\frac{dH}{dt}$ is the heat flow rate.

In the phenomenological model, the curing rate of the system can be expressed by the following equation:

(2) $$\frac{da}{dt}=k\left(T\right)*f\left(a\right)$$

where a is the curing degree; f(a) is a function of a, which is determined by the curing mechanism of the system; and k(T) is the curing rate constant that can be calculated by Arrhenius equation as follows:

(3) $$k\left(T\right)=A*exp\left(-\frac{Ea}{RT}\right)$$

where A is the pre-exponential factor; R is the gas constant; $E_{a}$ is the activation energy; and T is the absolute temperature.

Several reaction models have been reported [7,8,9,10]. In the present work, an *n*-order model as follows was used:

(4) $$\frac{d\alpha }{\mathrm{dt}}=k\left(T\right){\left(1-\alpha \right)}^{n}$$

where n is the reaction order. Therefore, the curing kinetic equation of the H_6_XDI/BES system can be expressed as:

(5) $$\frac{d\alpha }{dt}=A*\exp\left(-\frac{E_{a}}{RT}\right)*{\left(1-\alpha \right)}^{n}$$

The logarithmic form of Equation (5) is:

(6) $$ln\;\frac{d\alpha }{dt}=lnA+n*\ln\left(1-\alpha \right)-\frac{E_{a}}{RT}$$

Integration of Equation (6) under isothermal conditions leads to the following equation:

(7) $$\alpha =1-{\left[1-\left(1-n\right)*A*t*\exp\left(-\frac{E_{a}}{\mathrm{RT}}\right)\right]}^{\frac{1}{1-n}}$$

For the H_6_XDI/BES system, the Kissinger equation [11], in combination with the Crane equation [12], was used to obtain the value of parameters in the curing kinetic equations. The advantages of these equations include convenient fitting, high accuracy, and low dependence on the data quality of DSC. The Kissinger equation can be expressed as follows:

(8) $$ln\frac{\beta }{T_{p}^{2}}=ln\frac{AR}{E_{a}}-\frac{E_{a}}{RT_{p}}$$

where *β* is the heating rate during DSC testing, and $T_{p}$ is the peak temperature of the DSC curve.

**Figure 2:**
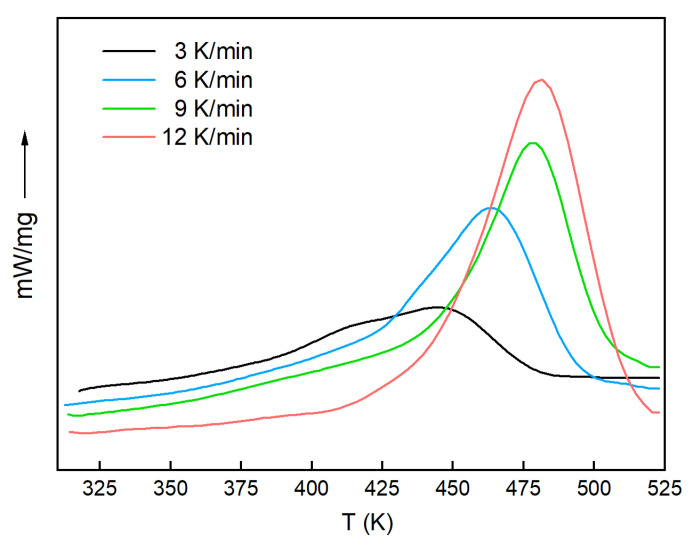
The DSC curves at different heating rates.

**Figure 3:**
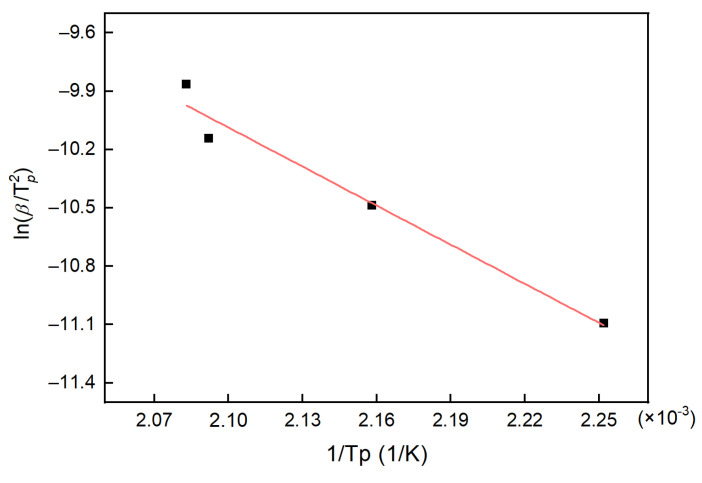
The fitting line based on the Kissinger equation.

The DSC curves of the system obtained at different heating rates are shown in Figure 2. The DSC results were analyzed, and the data are listed in Table 1. From the DSC curves and data, it was found that there was only one peak temperature for the H_6_XDI/BES system. With the increase in heating rate, the peak shifted to a higher temperature, due to heat-release hysteresis of the system. The data in Table 1 were fitted according to Equation (8), the result of which is shown in Figure 3.

Based on the fitting results in Figure 3, the activation energy of the H_6_XDI/BES system was calculated as $E_{a}\;$= 55.668 kJ/mol. The intercept of the fitting line was 3.9746, and the A value for the system equals 356,600.

The reaction order n of the system could be determined using the Crane equation as follows:

(9) $$\frac{\mathrm{dln}\beta }{\;d\left(\frac{1}{T_{p}}\right)}=-\left(\frac{E_{a}}{\mathrm{nR}}+2T_{p}\right)$$

As the peak temperature T_P_ was much smaller than the E_a_, Equation (9) could be transformed into the following equation:

(10) $$\frac{dln\beta }{\;d\left(\frac{1}{T_{p}}\right)}=-\frac{E_{a}}{nR}$$

**Figure 4:**
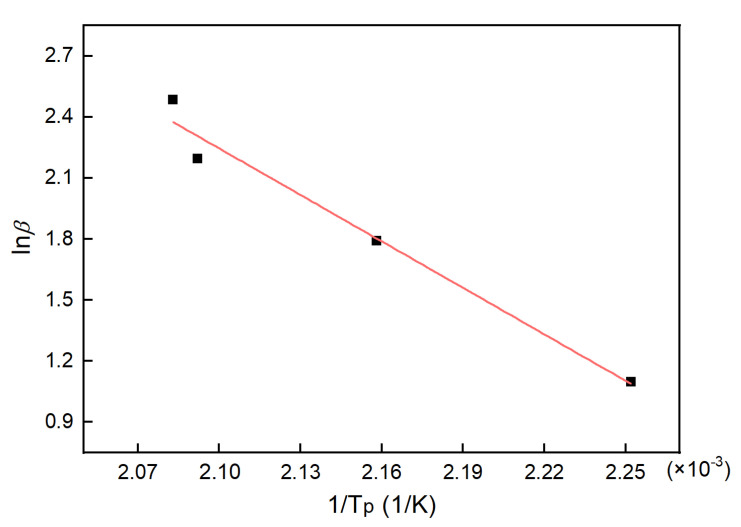
The fitting line according to the Crane equation.

The DSC data listed in Table 1 were analyzed using Equation (10), the results of which are shown in Figure 4. The n value for the system was thus calculated as 0.88.

The obtained values of n, E_a_, and A were put into Equation (5), giving the expression of the phenomenological model for the system as follows:

(11) $$\frac{d\alpha }{dt}=356600*\exp\left(-\frac{55668}{RT}\right)*{\left(1-\alpha \right)}^{0.88}$$

Integrating Equation (11) resulted in the following equation:

(12) $$\alpha =1-{\left[1-42792*t*\exp\left(-\frac{55668}{RT}\right)\right]}^{8.33}$$

**Figure 5:**
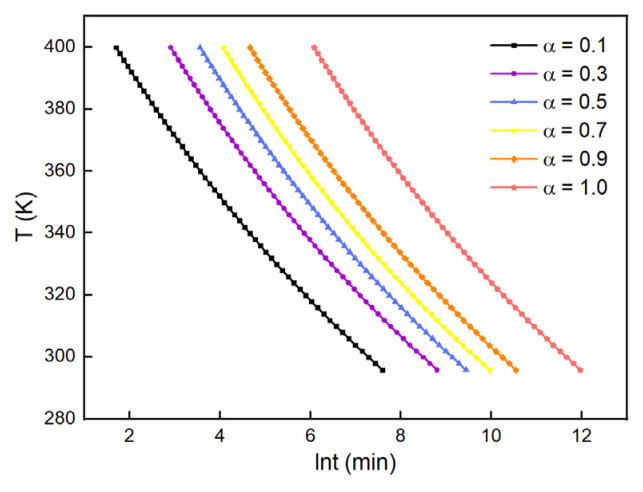
Iso–curing curves for the H_6_XDI/BES system.

Several degrees of cure values were put into Equation (12) to obtain the relationship between T and t at the fixed degree of cure. The relationship is depicted in Figure 5.

It can be seen in Figure 5 that more time was needed to increase the degree of cure with the curing process at the same temperature. At the initial stage of curing, the viscosity of the system was low at the relatively low crosslinking. The motion of molecules was rapid and it was more probable for the small molecules to react with each other, leading to the quick increase of cure degree. As the cure reaction proceeded, the increase in cure degree slowed down, since the crosslinked polymer dominated the system and the viscosity increased significantly. Through analyzing the iso–curing curves of the H_6_XDI/BES system, the time needed to achieve a certain degree of cure at a certain temperature could be obtained.

**Figure 6:**
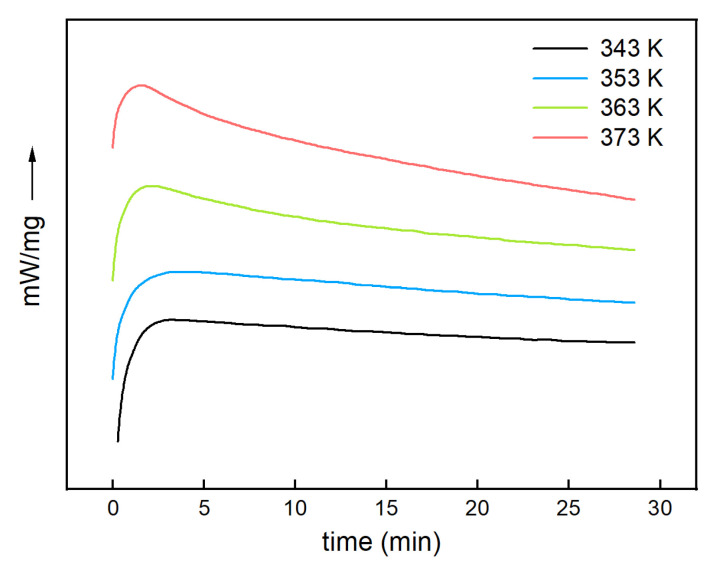
DSC curves obtained after 25 min incubation at different temperatures.

To confirm that the *n*-order model applied to the H_6_XDI/BES system, isothermal DSC testing was performed for the system at 343 K, 353 K, 363 K, and 373 K, respectively, for 25 min. The DSC curves are shown in Figure 6. The DSC curve became sharper with the increase in curing temperature. The cure reaction rate was highest at the initial stage of the reaction, in accordance with the *n*-order model.

#### 3.1.3. Determination of Glass Transition Curve

During the curing process, the residual enthalpy of the resin system decreased with increasing curing degrees. The residual enthalpy after the resin was cured various times at a temperature was measured according to DSC data. The curing degree α and the glass transition temperature Tg under different conditions could be calculated by analyzing the DSC data. According to the DiBenedetto empirical equation [13], α is related to Tg by the following equations:

(13) $$Tg=Tg_{0}+\frac{\lambda \alpha \left(Tg_{\infty }-Tg_{0}\right)}{1-\left(1-\lambda \right)\alpha }$$

and:

(14) $$\frac{1}{Tg-Tg_{0}}=\frac{1}{\alpha }*\frac{1}{\lambda \left(Tg_{\infty }-Tg_{0}\right)}-\frac{1-\lambda }{\lambda \left(Tg_{\infty }-Tg_{0}\right)}$$

At a constant temperature, the degree of cure increased with prolonged time. The residual enthalpy of the cured system was used to calculate the degree of cure, according to the following equation:

(15) $$\alpha \left(T,t\right)=1-\frac{\Delta H_{res}}{\Delta H_{total}}$$

where $\Delta H_{res}$ is the residual enthalpy, obtained from the area of peaks in the isothermal DSC curve.

**Figure 7:**
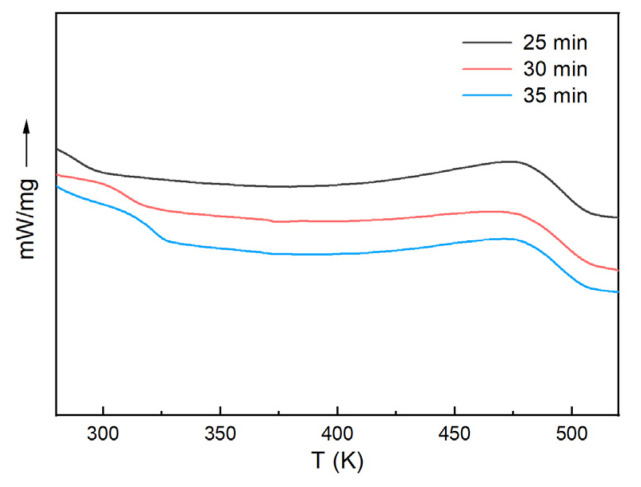
DSC curves of the H_6_XDI/BES system after incubation at 423 K for different times.

An isothermal DSC test was performed for the H_6_XDI/BES system at 423 K, and then the sample was subjected to DSC scanning from 273 K to 523 K at a heating rate of 10 K/min. The results are shown in Figure 7.

Figure 7 indicates that the area of the exothermic peaks decreased with the increase in cure time. The residual enthalpy decreased, and the glass transition temperature increased with increasing conversion, characteristic of the H_6_XDI/BES system. There was a baseline shift in all the curves at a temperature below 323 K. The corresponding temperature was lower than the ${\mathrm{Tg}}_{\infty }$, indicating that the curing of the system was incomplete. This was also reflected by the exothermic peak at 473 K. The isothermal DSC curves were analyzed according to Equation (15), the results of which are listed in Table 2.

**Figure 8:**
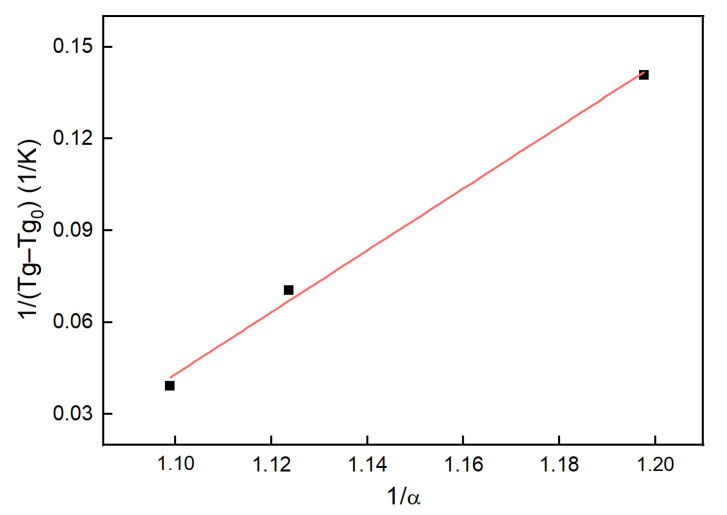
The fitting line based on the DiBenedetto equation.

The data in Table 2 along with ${\mathrm{Tg}}_{0}$ and ${\mathrm{Tg}}_{\infty }\;$ were fitted using Equation (14), giving the fitting line as shown in Figure 8.

The slope of the fitting line was 1.0110, and the λ in the Dibenedetto equation was 0.018. Then, the values of λ and α were put into Equation (14), giving the relationship between the glass transition temperature and temperature or time:

(16) $$t=\frac{1-{\left(\frac{6.432-0.0183{*T}_{g}}{T_{g}-294.8}\right)}^{0.12}}{42792*\exp\left(\frac{-6695.78}{T}\right)}$$

**Figure 9:**
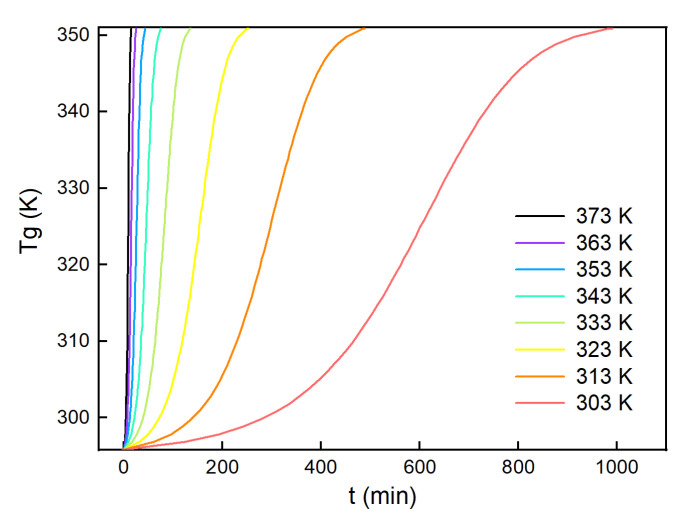
Relationship between glass transition temperature and time at constant temperatures.

The glass transition temperature was plotted against time at temperatures ranging from 303 to 373 K, which is shown in Figure 9. It can be seen that Tg became closer to ${\mathrm{Tg}}_{\infty }$ with prolonged time at each temperature. At t = 0, the glass transition temperature was close to ${\mathrm{Tg}}_{0}$. At low temperatures, Tg increased slowly. When the temperature increased beyond 323 K, Tg rapidly increased with time.

**Figure 10:**
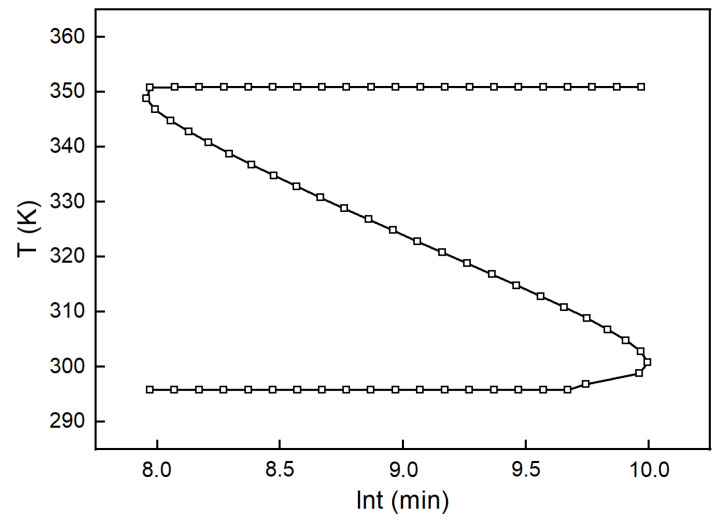
Glass transition curve of the H_6_XDI/BES system.

If ${\;T}_{g}$ was replaced by T, Equation (16) reflected the relationship between T and t. A plot based on this gave the glass transition curve, as shown in Figure 10. It is clear that the Tg of the H_6_XDI/BES system approached ${\mathrm{Tg}}_{0}\;$ with the increase in cure time and curing temperature. After the glass transition occurred, the curing reaction rate decreased significantly, because the motion of molecules was subjected to much greater hindrance.

#### 3.1.4. Determination of Gelation Curve

It was assumed that there is only one activation energy $E_{a}\;$ in the curing reaction of the resin system [4,14]. The curing reaction kinetic equation was integrated from α = 0 to α = $\alpha_{\mathrm{gel}}$, giving the following equation:

(17) $$\mathrm{lnt}\left(t_{\mathrm{gel}}\right)=\ln\left(\frac{\int_{0}^{\alpha_{\mathrm{gel}}}\frac{d\alpha }{f\left(\alpha \right)}}{A_{0}}\right)+\frac{\mathrm{Ea}}{\mathrm{RT}}$$

where the first term on the right is a constant and can be expressed by B:

(18) $$\ln\left(t_{\mathrm{gel}}\right)=B+\frac{\mathrm{Ea}}{\mathrm{RT}}$$

**Figure 11:**
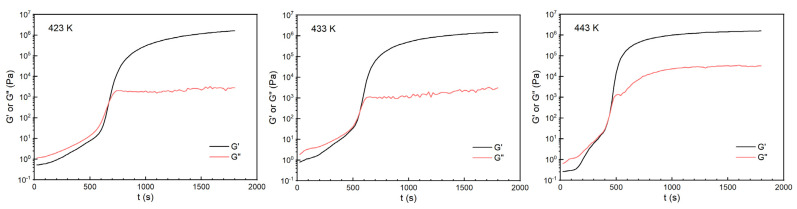
Changes in G’ and G” with time for the H_6_XDI/BES system at different temperatures.

Rheological tests were performed for the H_6_XDI/BES system, and the gelation times at 423 K, 433 K, and 443 K, respectively, were determined. The resultant rheological curves are shown in Figure 11.

The crossover of G’ and G” was regarded as the gel point of the system. The gelation time at different temperatures is listed in Table 3.

**Figure 12:**
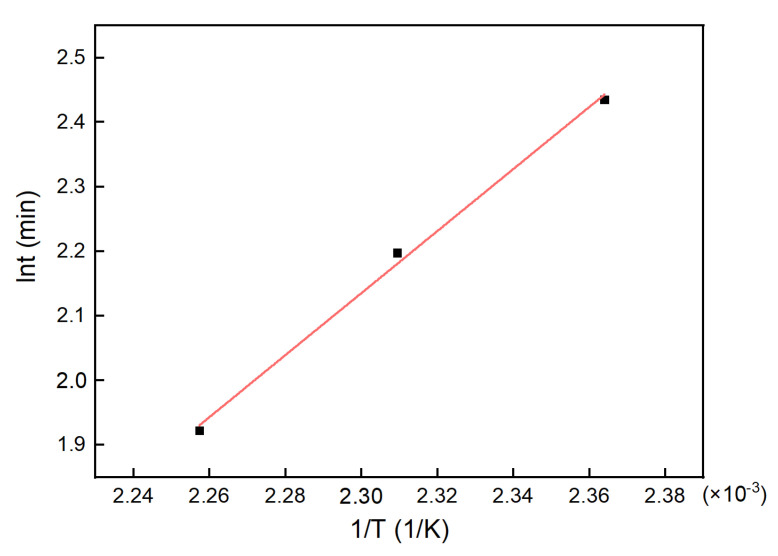
The fitting line for the gelation time of the H_6_XDI/BES system.

From Table 3, it can be seen that the gelation time decreased with the increase in cure time. According to Figure 11, the mobility of the system decreased dramatically at the point of gelation. The data in Table 3 were fitted using Equation (18), giving the results as depicted in Figure 12.

The parameters of the gelation model were calculated based on linear fitting. The slope of the fitting line gave ${\;E}_{\mathrm{gel}}=39.951\;\mathrm{kJ}/\mathrm{mol}$ and the intercept gave B = −8.917. These parameters were put into Equation (18), giving the equation correlating the gelation time ${\;t}_{\mathrm{gel}\;}$ with the temperature:

(19) $$lnt_{gel}=-8.917+\frac{4805.3182}{T}$$

**Figure 13:**
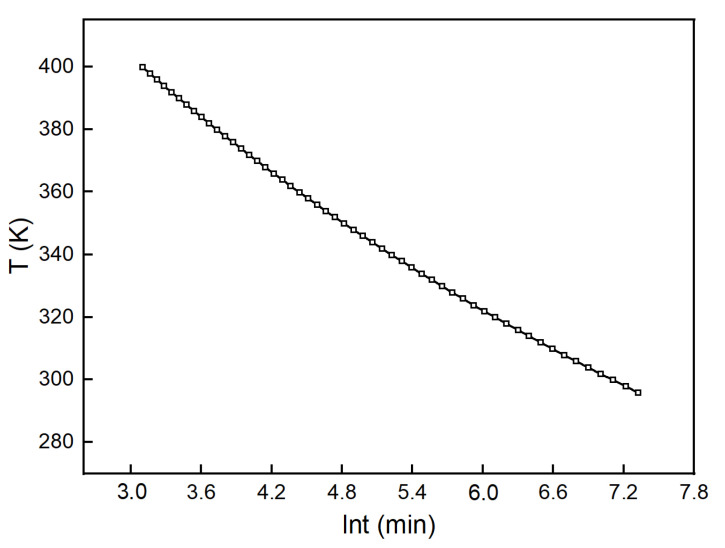
Gelation curve of the H_6_XDI/BES system.

Gelation curves could be obtained based on Equation (19), as shown in Figure 13. The chemical and physical changes in the resin system during the curing process can be reflected by the gelation curve. The gelation curve provides information on the reaction rate of the system during the cure reaction.

#### 3.1.5. Construction of TTT Diagram

**Figure 14:**
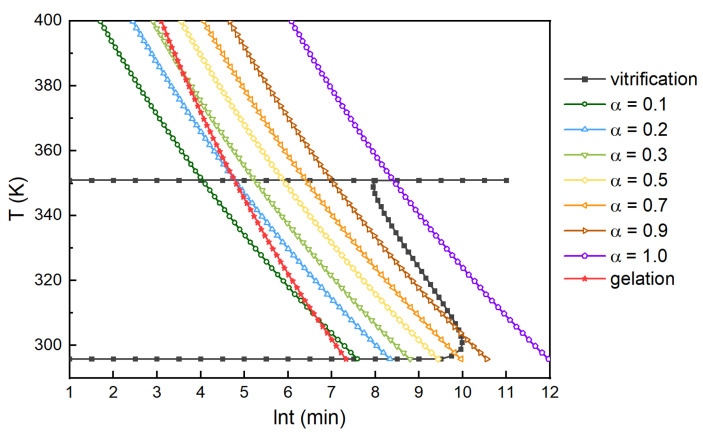
TTT diagram of the H_6_XDI/BES system.

The ${\mathrm{Tg}}_{0},{\;\mathrm{Tg}}_{\infty }$, curing curve, glass transition curve, and gelation curve were plotted in a diagram, taking lnt as the X axis and T as the Y axis. A TTT diagram of the H_6_XDI/BES system was constructed and is shown in Figure 14. It is clear that ${\mathrm{Tg}}_{0}=295.8\;K$ and ${\mathrm{Tg}}_{\infty }=350.9\;K$. The reaction rate was fairly low in the low-temperature region. Beyond the gelation point, the degree of cure increased significantly, and the time needed to achieve glass transition was longer. When the degree of cure increased to 0.9, its increase became extremely slow, which was in accordance with the results after the glass transition occurred. It can be concluded that prolonging cure time favors an increase in the degree of cure when it grows to 0.9. The TTT diagram is meaningful for optimizing the curing processing of the resin system.

### 3.2. Construction of TTT-η Diagram

A double Arrhenius equation with six parameters was used to describe the viscosity changes in the system:

(20) $$ln\eta =lnk_{1}+Tk_{2}+ln\eta_{\infty }+\frac{\Delta E_{\eta }}{RT}+t*K_{\infty }exp\left(-\frac{\Delta E_{a}}{RT}\right)$$

The six parameters could be obtained by the following equations:

(21) $$\;\frac{\eta_{t}}{{\;\eta }_{0}}=A_{0}\exp\left(\mathrm{Kt}\right)$$

(22) $${\ln\eta }_{0}={\ln\eta }_{\infty }+\frac{E_{\eta }}{\mathrm{RT}}$$

(23) $$\mathrm{lnK}={\mathrm{lnK}}_{\infty }-\frac{E_{a}}{\mathrm{RT}}$$

(24) $${\mathrm{lnA}}_{0}={\mathrm{lnk}}_{1}+k_{2}*T$$

where A_0_ is the pre-exponential factor; K is the reaction rate constant; $\eta_{\infty }\;\mathrm{and}\;K_{\infty }$ are pre-exponential factors for the Arrhenius equation; k_1_ and k_2_ are coefficients for A_0_; $\;E_{\eta }$ is the flow activation energy; and $E_{a}$ is the curing reaction activation energy.

**Figure 15:**
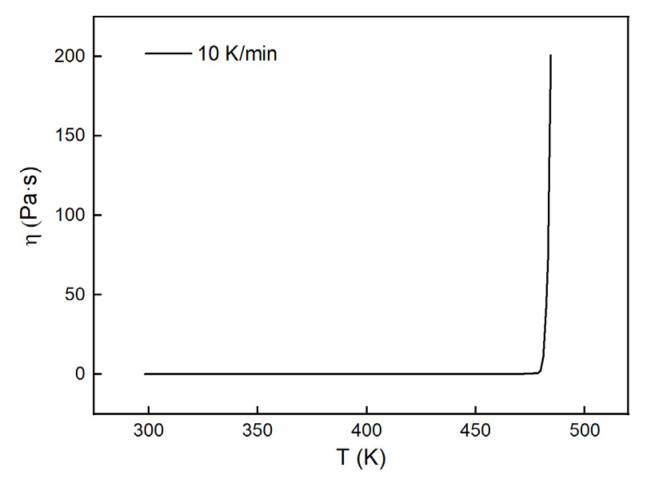
Viscosity changes with the temperature of the H_6_XDI/BES system.

Viscosity measurements were performed for the H_6_XDI/BES system at a heating rate of 10 K/min, the results of which are shown in Figure 15.

**Figure 16:**
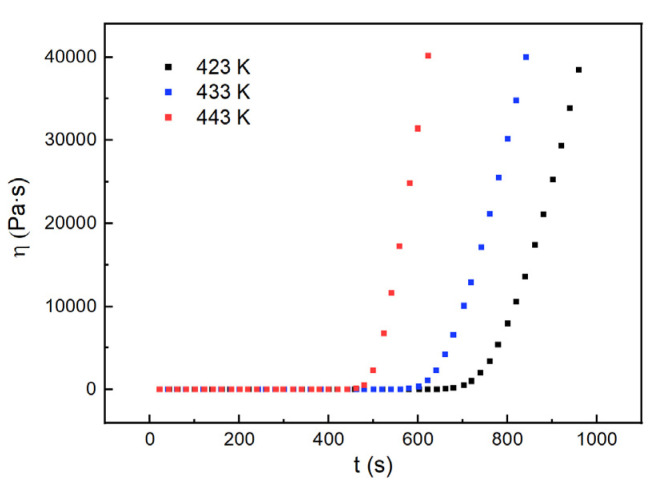
The viscosity of the H_6_XDI/BES system with time at constant temperatures.

Three temperatures of 423 K, 433 K, and 443 K at which the viscosity changed slightly were chosen to measure the viscosity of the H_6_XDI/BES system, the results of which are shown in Figure 16.

#### 3.2.1. Fitting of the Initial Viscosity η_0_

The initial viscosity values of the H_6_XDI/BES system at different temperatures are listed in Table 4.

**Figure 17:**
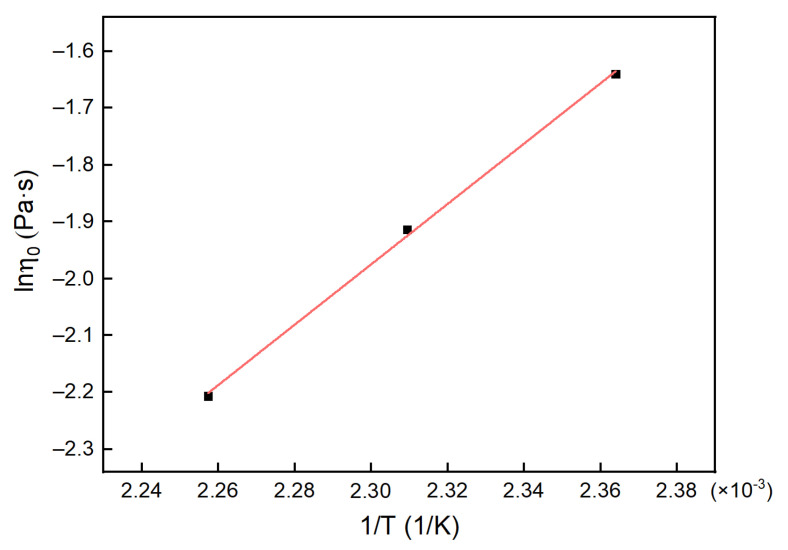
The fitting line for the *η*_0_ of the H_6_XDI/BES system.

The $\eta_{0}$ values were put into Equation (22), and the calculated results are shown in Figure 17.

Entering the data from Figure 17 into Equation (21), we obtained $E_{\eta }$ = 44.137 kJ/mol and $\eta_{\infty }$ = 3.539 × 10^−7^. The $\eta_{0}$ of the system was expressed as follows:

(25) $$ln\eta_{0}=-14.8544+\frac{5308.81261}{T}$$

#### 3.2.2. Fitting for the Reaction Rate Constant K and the Pre-Exponential Factor A_0_

**Figure 18:**
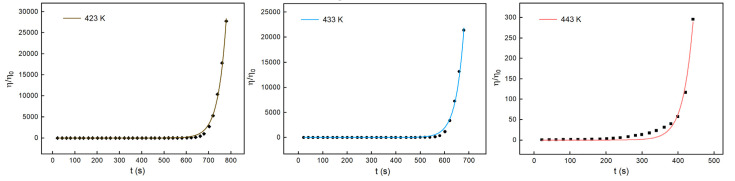
Changes in the relative viscosity with time at different temperatures.

**Figure 19:**
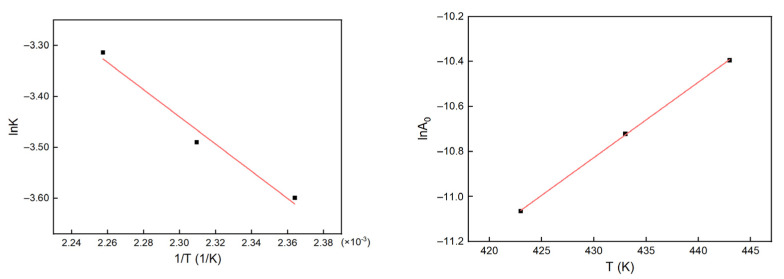
Fitting lines for  K  and ${\;A}_{0}$.

The relative viscosity at different temperatures was calculated and fitted based on Equation (21), the results of which are shown in Figure 18. The results of fitting are listed in Table 5. The data in Table 5 were calculated using Equations (23) and (24), the results of which are shown in Figure 19.

Through analyzing the fitting line for K, we obtained E_a_ = 22.201 kJ/mol and $K_{\infty }=14.9064$, and K was related to temperature by Equation (26). By analyzing the fitting line for $A_{0}$, we obtained k_1_ = 1.07566 × 10^−11^ and k_2_ = −0.03355, and the A_0_ was expressed by Equation (27).

(26) $$\mathrm{lnK}=2.70179-\frac{2670.3564}{T}$$

(27) $${\mathrm{lnA}}_{0}=-25.2555+0.03355*\mathrm{Ts}$$

#### 3.2.3. Construction and Verification of Rheological Model

Equations (25)–(27) were substituted into Equation (20), and the rheological modeling equation was obtained as shown in Equation (28):

(28) $$\eta =3.806*10^{-18}*exp\left[\frac{5308.8126}{T}-0.034*T+t*14.9064*\exp\left(\frac{-2670.3564}{T}\right)\right]$$

**Figure 20:**
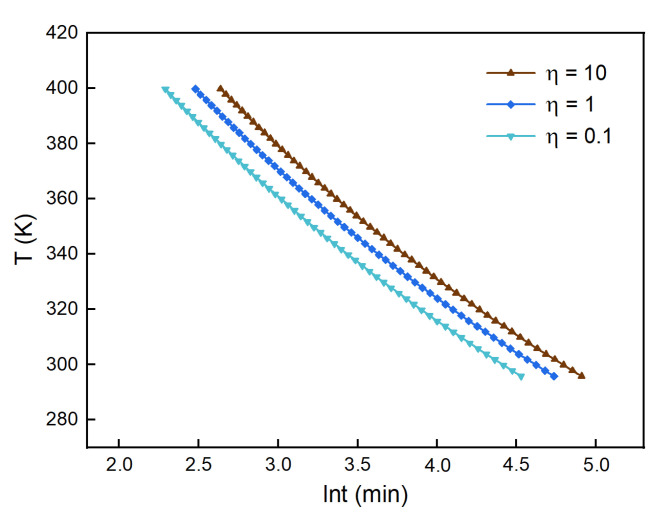
Iso–viscosity curves for the H_6_XDI/BES system.

If the viscosity was fixed, Equation (28) correlated the temperature and time, based on which the iso–viscosity curves for the system were obtained as shown in Figure 20. The viscosity changes in a resin system with the increase in cure time or curing temperature can be reflected by the viscosity curves. With the cure proceeding, the viscosity of the system gradually increased, and the increasing trend grew. This had instructive value for the technical design of our polyurethane system.

**Figure 21:**
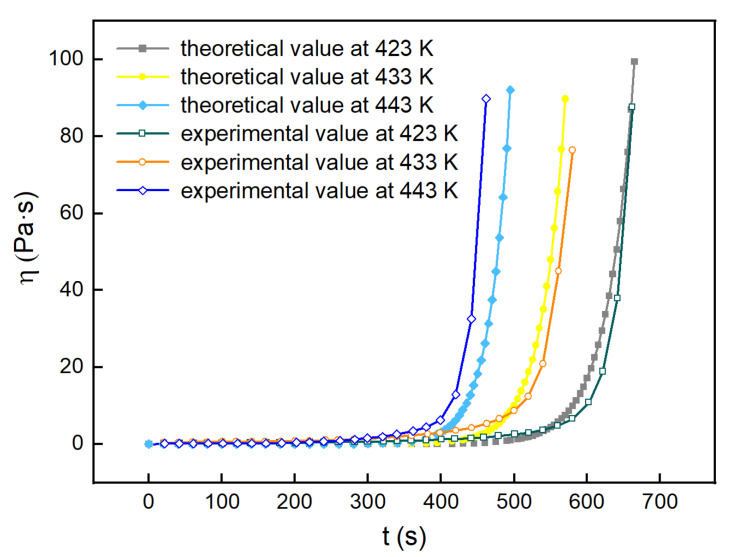
Theoretical and experimental viscosity curves of the H_6_XDI/BES system at different temperatures.

The experimental viscosity values measured at 423 K, 433 K, and 443 K were compared with the theoretical viscosity based on Equation (28), the results of which are shown in Figure 21.

As seen from Figure 21, in the region of low viscosity, the disparity between the experimental viscosity and the theoretical viscosity was small, while in the region of high viscosity, the disparity was larger. There were a variety of factors that influenced the viscosity of the system, and temperature and time were the primary factors considered. As the curing reaction proceeded to a certain degree, the curing reaction became complex, with the factors working together. The region where the experimental viscosity differed most greatly from the theoretical viscosity was located in the vicinity of the gelation point. At this point, the resin almost lost fluidity. Therefore, the viscosity model developed in this work is useful for the study of H_6_XDI/BES system.

#### 3.2.4. TTT-η diagram of H_6_XDI/BES System

**Figure 22:**
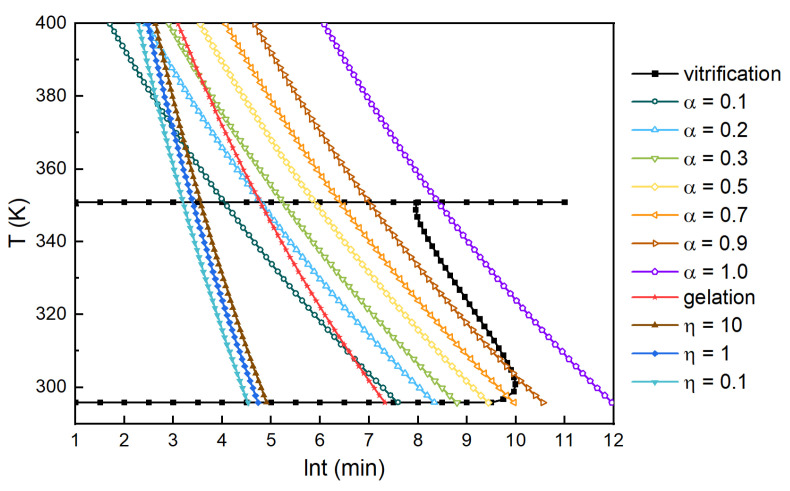
TTT-η diagram of the H_6_XDI/BES system.

The viscosity curves were added based on the TTT diagram, giving a TTT diagram of the H_6_XDI/BES system, as shown in Figure 22. From the TTT-η diagram, the viscosity of the resin system, as well as whether glass transition or gelation occurred, could be observed. The state of the resin system could thus be monitored and help to guide the processing parameters. Especially for composite materials, the TTT-η diagram can provide a useful platform for the design and optimization of processing routes.

## 4. Conclusions

In our present work, the curing process of the H_6_XDI/BES system was studied using both DSC and rheological measurements. A TTT-η diagram for the system was constructed to provide a theoretical basis for the production technique of the polyurethane system. The cure kinetics of the system were investigated by DSC at varying temperatures. The kinetic parameters were calculated and the relationship between the curing degree and time was obtained. Based on the isothermal DSC and DiBenedetto equation, the relationship between glass transition temperature and the curing degree was obtained. The gelation time of the system at different temperatures was determined by rotational rheometry and was fitted to obtain the relationship between gelation time and gelation temperature, producing the TTT diagram of the system. Based on the viscosity model, a double Arrhenius equation was utilized to investigate the rheological behavior of the system, establishing a rheological model and verifying its validity. The viscosity model was introduced on the basis of the TTT diagram, giving a TTT-η diagram to guiding the processing technique. With regard to a production process, numerous key parameters of the curing system, such as the physical state, the curing degree, and the viscosity, in any conditions of time and temperature, could be inferred according to the TTT-η diagram in theory. The production process could be further designed or optimized based on the guidance of the inferred parameters and then a product with excellent performance could be produced.

## Figures and Tables

**Table 1:** DSC data at different heating rates.

*β* (K/min)	T_p_ (K)
3	444.1
6	463.3
9	478.1
12	480.1

**Table 2:** DSC data based on Figure 7.

Incubation Time	$T_{g}\;\left(K\right)$	$\Delta H_{res}\;(J/g)$	α
25 min	302.90	51.57	0.835
30 min	310.00	34.52	0.890
35 min	321.24	28.08	0.910

**Table 3:** Gelation time at different temperatures for the H_6_XDI/BES system.

Temperature (K)	Gelation Time (min)
423	11.416
433	9.040
443	6.800

**Table 4:** $\eta_{0}$ values of the H_6_XDI/BES system at different temperatures.

T (K)	$\eta_{0}(\mathrm{Pa}\cdot s)$
423	0.1939
433	0.1465
443	0.1100

**Table 5:** The values of K and $A_{0}$ at different temperatures.

T/K	K	$A_{0}$
423	0.02735	1.56517 × 10^−5^
433	0.03050	2.20617 × 10^−5^
443	0.03639	3.06199 × 10^−5^

## Data Availability

The data presented in this study are available on request from the corresponding author.
